# Zinc Supplementation Partially Reconstitutes Impaired Interferon-γ Production in the Elderly

**DOI:** 10.3390/ijms27021039

**Published:** 2026-01-20

**Authors:** Krisztina Olah, Johanna Zenk, Jana Jakobs, Thea Laurentius, Leo Cornelius Bollheimer, Lothar Rink

**Affiliations:** 1 Institute of Immunology, Faculty of Medicine, RWTH Aachen University Hospital, Pauwelsstraße 30, 52074 Aachen, Germany; krisztina.olah@rwth-aachen.de (K.O.); johanna.zenk@rwth-aachen.de (J.Z.); jjakobs@ukaachen.de (J.J.); 2 Department of Geriatric Medicine, Faculty of Medicine, RWTH Aachen University Hospital, Morillenhang 27, 52074 Aachen, Germany; thea.laurentius@uni-oldenburg.de (T.L.); cbollheimer@ukaachen.de (L.C.B.)

**Keywords:** aging, cytokines, IFN-γ, zinc, dietary supplements, zinc supplementation, proton pump inhibitors

## Abstract

Aging impacts immunity, zinc status, and overall health, with these factors being closely interconnected. Zinc is known to modulate protein expression and cytokine production, with new molecular mechanisms continuing to be identified. ZIP8 facilitates IFN-γ production by increasing the intracellular zinc levels; how zinc status in humans affects ZIP8 expression remains unclear. We assessed serum zinc, dietary zinc intake, proton pump inhibitor (PPI) use, phytohemagglutinin (PHA)-stimulated IFN-γ production, and ZIP8 protein expression in elderly hospitalized patients and young healthy controls. Compared to young adults, elderly participants exhibited lower zinc status and IFN-γ levels, with PPI use among the elderly correlating with zinc deficiency. Zinc-deficient elderly participants received zinc aspartate supplementation for approximately 7 days, resulting in increased serum zinc levels, IFN-γ production, and a trend toward increased ZIP8 expression; in participants taking PPIs, this increase reached statistical significance. Although we found no clear correlation between ZIP8 expression and zinc status, the observed response to supplementation warrants further investigation. These findings reinforce the relevance of zinc supplementation in the elderly, although further studies are needed to elucidate the precise mechanisms linking zinc status to IFN-γ production, particularly regarding the role of ZIP8 expression levels.

## 1. Introduction

The aging process poses significant challenges to the immune system, as it leads to the decline and dysregulation of multiple immune functions. Clinically, this presents as an increased propensity for infections, cancer, and autoimmune diseases and an impaired vaccination response in elderly patients [1,2,3]. The entirety of changes in the elderly immune system is broadly regarded as immunosenescence. Markers of immunosenescence include a decline in Th1 response and reduced lymphocyte counts [4,5,6]. Interferon (IFN)-γ is a key Th1 cytokine, critical for the response to infections and vaccines [7,8]. It is well established that the production of IFN-γ by lymphocytes of the elderly is significantly lower compared to healthy young adults [5]. In addition, it has been shown that IFN-γ production in response to immune stimulation is positively impacted by zinc [9,10,11].

Zinc is an essential trace element with a crucial role in the immune system. Zinc regulates the immune response by promoting regulatory T-cells [12] and the production of anti-inflammatory cytokines [11,13]. Zinc also promotes the polarization towards Th1 response [10]. Zinc deficiency is a common phenomenon in the elderly population—both in absolute values and relative to the young adult population [14]. Studies have shown a negative correlation between serum zinc and age—in animal models [15] and in healthy elderly people [16]. Lower dietary zinc intake [17], a decline in intestinal zinc absorption [15], and an increase in zinc excretion [15] are considered contributing factors to this deficiency. Chronic illness [18,19] and hospitalization [20] clearly correlate with lower zinc levels; however, the direction of causality remains unclear. Certain medications appear to negatively impact zinc levels [21], which is especially concerning in the elderly population, where the regular use of multiple medications is common [22].

Proton pump inhibitors (PPIs), one of the most commonly prescribed medication groups in the elderly, have been shown to impair nutrient absorption [23], increase the risk of infections [23,24,25,26], and reduce IFN-γ production in vitro [24,27] via suppression of the lysosomal zinc transporter ZIP8 [27], which has been implicated in zinc-dependent regulation of IFN-γ production [27,28,29]. IRF1 and IRF3 are transcription factors involved in the modulation of interferon responses and have been shown to respond to changes in zinc conditions [12,30,31].

We hypothesized that zinc deficiency in hospitalized elderly individuals is associated with reduced IFN-γ production, which could be partially restored by short-term zinc supplementation. As secondary objectives, we examined whether regular PPI use is associated with altered zinc status and IFN-γ production, and we exploratorily assessed the expression of ZIP8, IRF1, and IRF3 as potential molecular correlates of zinc-related and PPI-mediated alterations in IFN-γ production.

We report that short-term zinc supplementation results in a significant improvement in impaired zinc status and IFN-γ production in the hospitalized elderly. We show that regular PPI use affects the serum zinc concentration and dietary zinc intake score. However, no significant correlation between PPI intake and IFN-γ production was found, and our findings regarding molecular pathways warrant further research.

## 2. Results 

### 2.1. Study Cohort

**Figure 1:**
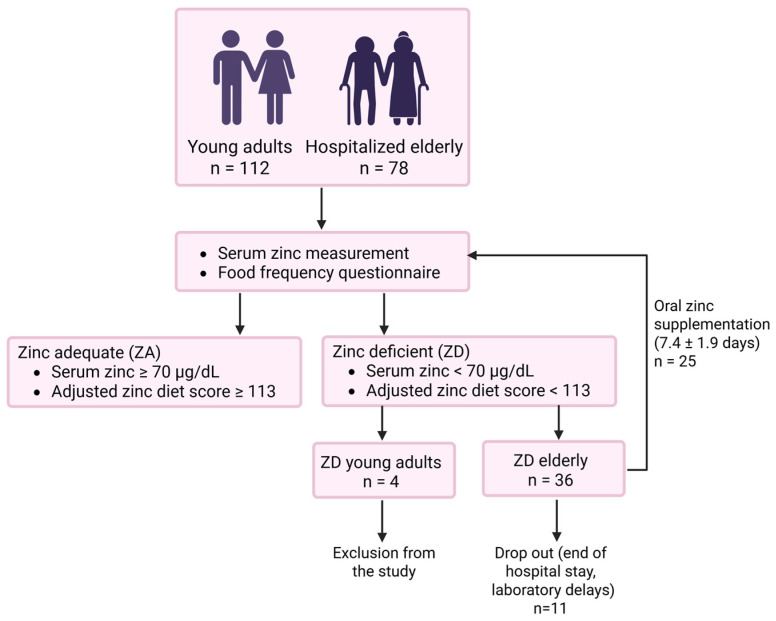
Study design. Zinc status was assessed by serum zinc concentration measurement and by the adjusted zinc diet score (AZDS) derived from the food frequency questionnaire (www.zink-app.de). Four zinc-deficient young adults were excluded from the study, while 36 zinc-deficient hospitalized elderly participants received daily oral zinc supplementation with Unizink^®^ 50 (10 mg zinc aspartate). Post-supplementation blood samples were planned approximately 7 days (7.4 ± 1.9 days) after initiation of supplementation. However, these samples were obtained from only 25 participants due to hospital discharges and laboratory-related delays. This figure was created in BioRender.

A total of 78 elderly hospitalized participants (mean age 82.2 ± 5.4 years, median age 83 years, age range 70–92 years) and 112 young healthy adults (mean age 24.3 ± 3.4 years, median age 24 years, age range 18–35), who served as the control group, were included in the study between April 2023 and April 2024. Consistent with previous studies [17], participants were considered zinc-deficient if they had an AZDS (adjusted dietary zinc score) below 113 points or serum zinc concentration below 70 μg/dL. Of the elderly participants, 36 (46%) met these criteria and received the zinc supplement Unizink^®^ 50 (Köhler Pharma GmbH, Alsbach-Hähnlein, Germany), as clinically indicated. Post-supplementation blood samples were collected approximately 7 days (7.4 ± 1.9 days) after supplementation in 25 participants. Due to hospital discharge or laboratory delays, post-supplementation samples of 11 participants were not obtained. In the young control group, 4 participants (4%) were zinc-deficient and were therefore excluded from further experiments. A schematic overview of the study design is provided in Figure 1.

### 2.2. Zinc Status

**Figure 2:**
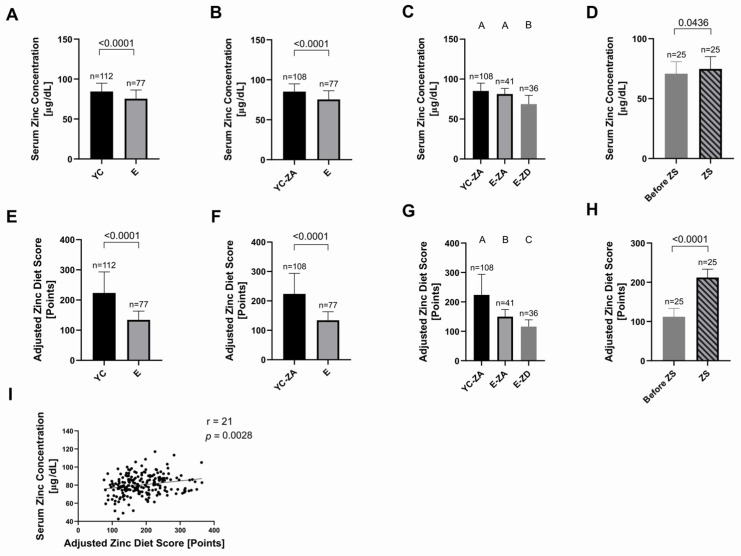
Markers of zinc status. Serum zinc concentration measured by atomic absorption spectroscopy (AAS), obtained from different groups: (**A**) young adults as a whole (YC, n = 112) and elderly cohort (E, n = 77)—YA vs. E: *p* < 0.0001; (**B**) zinc-adequate young controls (YC-ZA, n = 108) and E (n = 77)—YC-ZA vs. E: *p* < 0.0001; (**C**) YC-ZA (n = 108), zinc-adequate elderly (E-ZA, n = 41), and zinc-deficient elderly (E-ZD, n = 36)—YC-ZA vs. E-ZA: *p* = 0.2212; YC-ZA vs. E-ZD: *p* < 0.0001; E-ZA vs. E-ZD: *p* < 0.0001; (**D**) E-ZD (n = 25) who received daily oral supplementation with 10 mg of zinc aspartate (ZS) for approximately 7 days (7.4 ± 1.9 days)—before ZS vs. ZS: *p* = 0.0436. Adjusted zinc diet score (AZDS) calculated via food frequency questionnaire (www.zink-app.de), reflecting the dietary zinc intake, obtained from different groups: (**E**) young adults as a whole (YC, n = 112) and elderly cohort (E, n = 77)—YC vs. E: *p* < 0.0001; (**F**) zinc-adequate young controls (YC-ZA, n = 108) and E (n = 77)—YC-ZA vs. E: *p* < 0.0001; (**G**) YC-ZA (n = 108), zinc-adequate elderly (E-ZA, n = 41), and zinc-deficient elderly (E-ZD, n = 36)—YC vs. E-ZA: *p* < 0.0001; YC-ZA vs. E-ZD: *p* < 0.0001; E-ZA vs. E-ZD: *p* = 0.0058; (**H**) E-ZD (n = 25) who received daily oral supplementation with 10 mg of zinc aspartate (ZS) for approximately 7 days (7.4 ± 1.9 days)—before ZS vs. ZS: *p* < 0.0001. (**I**) Correlation between AZDS and serum zinc concentration (n = 207, *r* = 0.21, *p* = 0.0028) in all available samples. Data in (**A**–**H**) are shown as means + SD. An outlier test (ROUT 1%) was performed on all data sets, and outliers were removed (serum zinc concentration: E n = 1; E-ZA n = 1. AZDS: E n = 1; E-ZA n = 1; AZDS in all samples n = 3; serum zinc concentration in all samples n = 1). Data sets in (**A**–**H**) were tested by the D’Agostino and Pearson test for normality. Data sets in (**A**–**C**,**E**–**G**) were considered unpaired and data in (**D**,**H**) paired, since the same subjects were analyzed before and after supplementation. For unpaired non-normally distributed data, we used the Mann–Whitney test for comparing two data sets (**A**,**B**,**E**,**F**) and the Kruskal–Wallis test followed by Dunn’s multiple comparisons test for multiple data sets (**C**,**G**). For paired normally distributed data, the paired *t* test was used (**D**). For paired non-normally distributed data, the Wilcoxon test was used (**H**). Correlation in (**I**) was assessed using the Spearman correlation. Statistical significance is defined as *p* < 0.05. Means not sharing any letter in (**C**,**G**) are significantly different (*p* < 0.05).

We observed a significantly lower serum zinc concentration in the elderly group as a whole compared to young adults (*p* < 0.0001) (Figure 2A,B). Zinc-adequate and zinc-deficient elderly groups differed from each other in a significant way (*p* < 0.0001) (Figure 2C). Zinc-deficient elderly participants had a significantly lower serum zinc concentration compared to the young control group (*p* < 0.0001) (Figure 2C). However, the difference between the young control and the zinc-adequate elderly group was not statistically significant (*p* = 0.2212) (Figure 2C).

Supplementation of zinc-deficient patients with Unizink^®^ 50 led to a significant increase in serum zinc concentration (*p* = 0.0436) (Figure 2D), confirming the results from other zinc supplementation studies in our group [17,31].

The adjusted zinc diet score (AZDS) is a tool that has proved to be a valuable method for determining zinc status based on dietary information [32]. We show a significantly higher zinc diet score in the young adults group compared to the hospitalized elderly (*p* < 0.0001) (Figure 2E,F). It is worth noting that the questionnaire reflects dietary habits during the last six weeks, irrespective of hospitalization. To check whether the low scores of zinc-deficient elderly patients led to this difference between the old and the young, we conducted an ANOVA of zinc-adequate young controls and zinc-adequate and zinc-deficient elderly patients—the difference was significant both between zinc-adequate elderly patients and zinc-adequate young controls (*p* < 0.0001), and between the zinc-deficient elderly patients and zinc-adequate young controls (*p* < 0.0001) (Figure 2G); the AZDS of zinc-adequate elderly and zinc-deficient elderly patients also differed significantly (*p* = 0.0058) (Figure 2G). This finding points to the difference between young controls and elderly patients as a whole—irrespective of the zinc status of the elderly.

The zinc diet score increased significantly after zinc supplementation compared to the values determined before the supplementation (*p* < 0.0001) (Figure 2H). It could be argued that this is a test artifact since as soon as daily zinc supplementation with Unizink^®^ 50 is entered into the score calculator, the score automatically increases by 100 points. However, when considering all samples—including post-supplementation samples—the correlation between serum zinc concentration and the AZDS remains highly significant (*p* = 0.0014), implying once again that AZDS is a reliable tool for determining zinc status (Figure 2I).

### 2.3. IFN-γ

IFN-γ production was measured in phytohemagglutinin (PHA)-L-stimulated whole-blood samples and PBMCs using ELISA.

**Figure 3:**
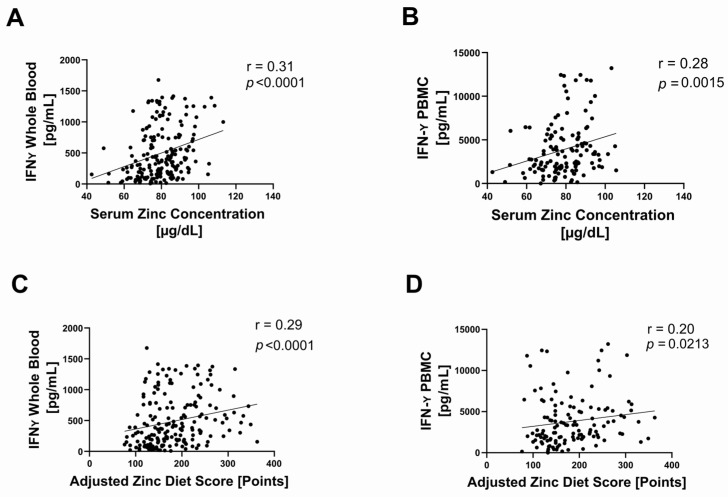
Correlations between markers of zinc status and IFN-γ production in all available samples using Spearman correlation. Correlation between serum zinc concentration (measured by atomic absorption spectroscopy) and IFN-γ production measured by ELISA after a 48 h stimulation with 2.5 μg/mL of PHA-L at 37 °C and 5% CO2 in (**A**) whole-blood samples (n = 185, r = 0.31, *p* < 0.0001) and in (**B**) isolated PBMCs (n = 128, r = 0.28, *p* = 0.0015). Correlation between adjusted zinc diet score (AZDS)—calculated via food frequency questionnaire (www.zink-app.de), reflecting the dietary zinc intake—and IFN-γ production in (**C**) whole-blood samples (n = 184, r = 0.29, *p* < 0.0001) and (**D**) isolated PBMCs (n = 128, r = 0.20, *p* = 0.0213). An outlier test (ROUT 1%) was performed on all data sets, and outliers were removed (serum zinc concentration in all samples n = 1; AZDS in all samples n = 3; IFN-γ whole-blood in all samples n = 24; IFN-γ PBMC in all samples n = 25). Statistical significance is defined as *p* < 0.05.

We found a significant positive correlation between serum zinc concentration and IFN-γ production in whole-blood samples (*p* < 0.0001) (Figure 3A) and in PBMCs (*p* = 0.0015) (Figure 3B). Likewise, AZDS showed significant positive correlations with IFN-γ production in whole-blood samples (*p* < 0.0001) (Figure 3C) and PBMCs (*p* = 0.0213) (Figure 3D).

**Figure 4:**
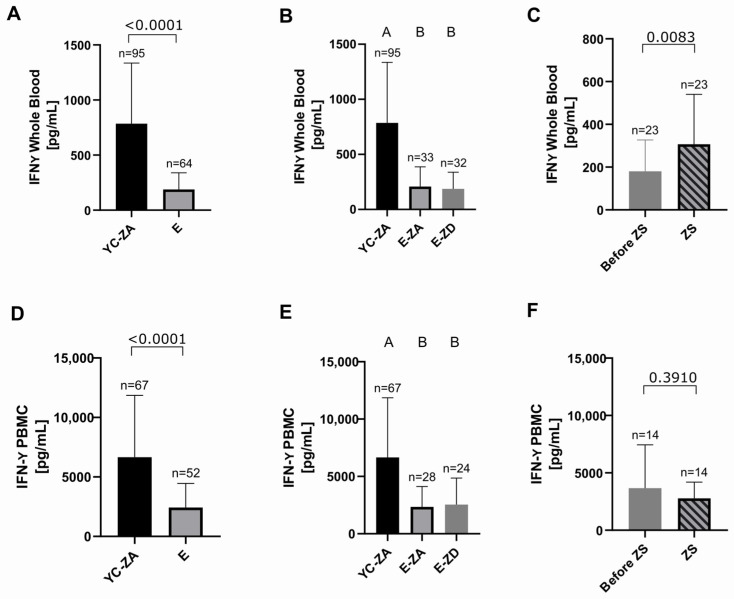
IFN-γ production measured by ELISA after 48 h of stimulation with 2.5 μg/mL of PHA-L at 37 °C and 5% CO_2_ in whole-blood samples, obtained from different groups: (**A**) zinc-adequate young controls (YC, n = 95) and elderly (E, n = 64)—YC vs. E: *p* < 0.0001; (**B**) YC (n = 95), zinc-adequate elderly (E-ZA, n = 33), and zinc-deficient elderly (E-ZD, n = 32)—YC vs. E-ZA: *p* < 0.0001; YC vs. E-ZD: *p* < 0.0001; E-ZA vs. E-ZD: *p* > 0.9999; (**C**) E-ZD (n = 25) who received daily oral supplementation with 10 mg of zinc aspartate (ZS) for approximately 7 days (7.4 ± 1.9 days)—before ZS vs. ZS: *p* = 0.0083. IFN-γ production measured by ELISA after 48 h of stimulation with 2.5 μg/mL of PHA-L at 37 °C and 5% CO_2_ in isolated PBMCs, obtained from different groups: (**D**) zinc-adequate young controls (YC, n = 67) and E (n = 52)—YC vs. E: *p* < 0.0001; (**E**) YC (n = 67), zinc-adequate elderly (E-ZA, n = 28), and zinc-deficient elderly (E-ZD, n = 24)—YC vs. E-ZA: *p* < 0.0001; YC vs. E-ZD: *p* = 0.0001; E-ZA vs. E-ZD: *p* > 0.9999; (**F**) E-ZD (n = 14) who received daily oral supplementation with 10 mg of zinc aspartate (ZS) for approximately 7 days (7.4 ± 1.9 days)—before ZS vs. ZS: *p* = 0.3910. Data are shown as means + SD. An outlier test (ROUT 1%) was performed on all data sets, and outliers were removed (IFN-γ whole blood: YC n = 13, E n = 13, E-ZA 8, E-ZD n = 4, before ZS n = 2, ZS n = 2, all samples n = 24; IFN-γ PBMC: YC n = 8, E n = 7, E-ZA n = 4, E-ZD n = 3, ZS n = 4, all samples n = 25). All data sets were tested by the D’Agostino and Pearson test for normality. Data sets in (**A**,**B**,**D**,**E**) were considered unpaired and data in (**C**,**F**) paired, since the same subjects were analyzed before and after supplementation. For unpaired non-normally distributed data, we used the Mann–Whitney test for comparing two data sets (**A**,**D**) and the Kruskal–Wallis test followed by Dunn’s multiple comparisons test for multiple data sets (**B**,**E**). For paired normally distributed data, the paired *t* test was used (**F**) and for paired non-normally distributed data, the Wilcoxon test was used (**C**). Statistical significance is defined as *p* < 0.05. Means not sharing any letter in (**B**,**E**) are significantly different (*p* < 0.05).

In accordance with previous evidence [5], we observed a significantly lower production of IFN-γ in elderly patients compared to the zinc-adequate young control group (*p* < 0.0001), both in whole blood (Figure 4A) and in PBMC samples (Figure 4D). Although the zinc-adequate and zinc-deficient elderly both show significantly lower IFN-γ production compared to the young control group, we could not show a significant difference in IFN-γ production when comparing the zinc-adequate and zinc-deficient elderly both in whole blood (Figure 4B) and PBMC samples (Figure 4E). This could imply that zinc status is not the only mechanism contributing to the production of IFN-γ. Different diseases, medications used, and other confounding factors could complicate the analysis of the impact of individual factors on cytokine production.

However, the statistically significant positive effect of the zinc supplementation in whole blood (*p* = 0.0083) (Figure 4C) is a promising first step toward understanding of zinc–cytokine connection in a heterogeneous human population. The same effect was not shown in PBMC samples (Figure 4F), likely reflecting the limitations of the PBMC culture model itself.

### 2.4. PPI Effect

**Figure 5:**
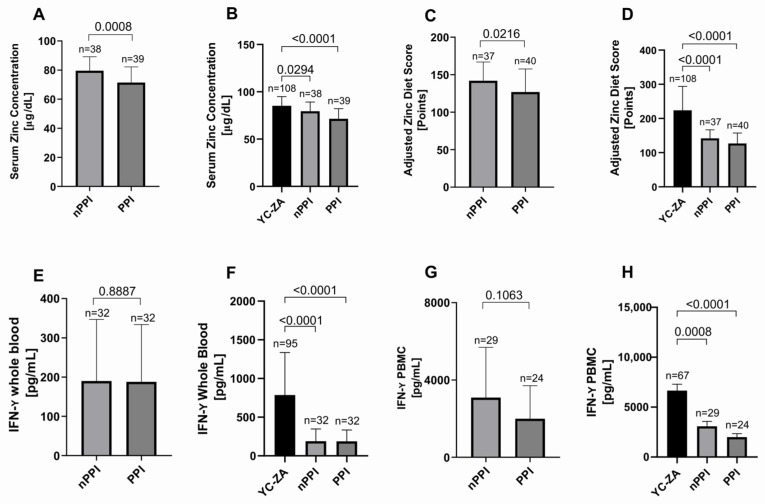
PPI effect. Serum zinc concentration measured by atomic absorption spectroscopy (AAS), obtained from different groups: (**A**) the elderly not taking PPIs (nPPI, n = 38) and those who do (PPI, n = 39)—nPPI vs. PPI: *p* = 0.0008; (**B**) YC-ZA (n = 108), nPPI (n = 38), and PPI (n = 39)—YC-ZA vs. nPPI: *p* = 0.0294; YC-ZA vs. PPI: *p* < 0.0001. Adjusted zinc diet score (AZDS) calculated via a food frequency questionnaire (www.zink-app.de), reflecting the dietary zinc intake, obtained from different groups: (**C**) the elderly not taking PPIs (nPPI, n = 37) and those who do (PPI, n = 40)—nPPI vs. PPI: *p* = 0.0206; (**D**) YC-ZA (n = 108), nPPI (n = 37), and PPI (n = 40)—YC-ZA vs. nPPI: *p* < 0.0001; YC vs. PPI: *p* < 0.0001. IFN-γ levels in whole-blood samples after 48 h of incubation with PHA-L at 37 °C and 5% CO_2_, measured by ELISA, obtained from different groups: (**E**) the elderly not taking PPIs (nPPI, n = 32) and those who do (PPI, n = 32)—nPPI vs. PPI: *p* = 0.8887; (**F**) YC (n = 95), nPPI (n = 32), and PPI (n = 32)—YC vs. nPPI: *p* < 0.0001; YC vs. PPI: *p* < 0.0001. IFN-γ levels in PBMCs after 48 h of incubation with PHA-L at 37 °C and 5% CO_2_, measured by ELISA, obtained from different groups: (**G**) the elderly not taking PPIs (n = 29) and those who do (PPI, n = 24)—nPPI vs. PPI: *p* = 0.1063; (**H**) YC (n = 67), nPPI (n = 29), and PPI (n = 24)—YC vs. nPPI: *p* = 0.0008; YC vs. PPI: *p* < 0.0001. All data are shown as means + SD. An outlier test (ROUT 1%) was performed on all data sets, and outliers were removed (serum zinc concentration: PPI n = 1; AZDS: nPPI n = 1; IFN-γ whole blood: nPPI n = 6, PPI n = 7; IFN-γ PBMC: nPPI n = 3, PPI n = 3). All data sets were tested by the D’Agostino and Pearson test for normality. All data sets were considered unpaired. For unpaired normally distributed data, an unpaired *t* test was used (**A**,**C**). For unpaired non-normally distributed data, we used the Mann–Whitney test for comparing two data sets (**E**,**G**), and the Kruskal–Wallis test followed by Dunn’s multiple comparisons test for multiple data sets (**B**,**D**,**F**,**H**). Statistical significance is defined as *p* < 0.05.

When comparing elderly participants based on their PPI intake, we observed a significantly lower serum zinc concentration in the elderly group taking PPIs (PPI group) compared to the elderly who do not (nPPI group) (*p* = 0.0008) (Figure 5A). Both the PPI and nPPI groups significantly differed from the young control group (*p* < 0.0001 and *p* = 0.0294, respectively) (Figure 5B).

To analyze whether the use of PPIs affects dietary zinc intake, we compared the AZDS of the PPI and nPPI groups. We showed a significantly lower AZDS in the PPI group (*p* = 0.0206) (Figure 5C). Both groups had a significantly lower AZDS compared to the zinc-adequate young control group (*p* < 0.0001 each) (Figure 5D).

Zinc deficiency occurred significantly more often in the PPI group (χ^2^ test, *p* = 0.0118). The relative risk was 1.86 (95% CI 1.14–3.20), suggesting that elderly people using PPIs had nearly twice the risk of zinc deficiency compared to non-users.

Regarding the production of IFN-γ, no significant differences were observed based on PPI intake in whole blood (*p* = 0.8887) (Figure 5E) or PBMC samples (*p* = 0.1063) (Figure 5G). However, both the nPPI and PPI groups significantly differed from the young zinc-adequate group in whole blood (*p* < 0.0001 each) (Figure 5F) and PBMC samples (*nPPI* vs. *YC*: *p* = 0.0008; PPI vs. YC-ZA: *p* < 0.0001) (Figure 5H). As mentioned previously, analyzing the effect of individual factors, such as PPI use, in a heterogeneous human population has its limitations. Further studies with larger sample sizes, adjusting for multiple confounders, are needed to fully understand the implications of PPI use for cytokine production.

### 2.5. ZIP8

**Figure 6:**
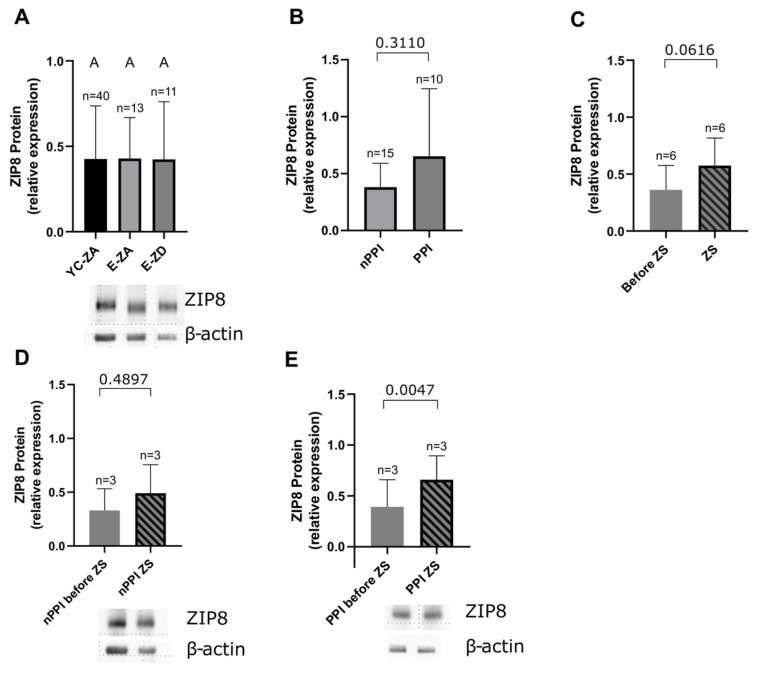
ZIP8 protein expression in unstimulated PBMCs, measured by Western blot, normalized to β-actin and to the reference sample in different groups: (**A**) zinc-adequate young controls (YC-ZA, n = 38), zinc-adequate elderly (E-ZA, n = 14), and zinc-deficient elderly (E-ZD, n = 11)—YC-ZA vs. ZA: *p* > 0.9999; YC vs. E-ZD: *p* > 0.9999; E-ZA vs. E-ZD: *p* > 0.9999; (**B**) the elderly not taking PPIs (nPPI, n = 15) and those who do (PPI, n = 10)—nPPI vs. PPI: *p* = 0.3110; (**C**) E-ZD (n = 6) who received daily oral supplementation with 10 mg of zinc aspartate (ZS) for approximately 7 days (7.4 ± 1.9 days)—before ZS vs. ZS: *p* = 0.0616; these were then separated into the nPPI (**D**) (n = 3, *p* = 0.4897) and PPI group (**E**) (n = 3, *p* = 0.0047) to assess the effect of zinc supplementation in these groups. Data are shown as means + SD. An outlier test (ROUT 1%) was performed on all data sets and outliers were removed (E-ZA n = 1). Data sets with n < 8 were tested by the Shapiro–Wilk test and data sets with n ≥ 8 by the D’Agostino and Pearson test for normality. Data sets in (**A**,**B**) were considered unpaired and data in (**C**–**E**) paired, since the same subjects were analyzed before and after supplementation. For unpaired non-normally distributed data, we used the Mann–Whitney test for comparing two data sets (**B**) and the Kruskal–Wallis test followed by Dunn’s multiple comparisons test for multiple data sets (**A**). For paired normally distributed data, the paired *t* test was used (**C**–**E**). Statistical significance is defined as *p* < 0.05. Means not sharing any letter in (**A**) are significantly different (*p* < 0.05). Representative Western blots are shown under (**A**,**D**,**E**).

With previous evidence that ZIP8 regulates intracellular zinc and thus IFN-γ production [27,28], we analyzed how its expression varies according to the zinc status of our participants. We found no significant differences between young controls, zinc-adequate elderly participants, and zinc-deficient elderly participants (*p* > 0.9999 in all comparisons) (Figure 6A). After zinc supplementation of zinc-deficient elderly participants, a trend toward increased ZIP8 expression was observed, albeit not reaching statistical significance (*p* = 0.0616) (Figure 6C). Once we stratified our elderly participants according to PPI intake, a significant increase in ZIP8 expression post supplementation was shown in the participants taking PPIs (PPI group) (n = 3, *p* = 0.0047) (Figure 6E), whereas the same effect in elderly participants not taking PPIs was not seen (nPPI group) (n = 3, *p* = 0.4897) (Figure 6D). Considering the small sample size, further studies with larger sample sizes are necessary to confirm or refute these findings. Overall, we observed no significant differences in the ZIP8 expression between PPI and nPPI groups (*p* = 0.3110) (Figure 6B).

### 2.6. IRF1 and IRF3

**Figure 7:**
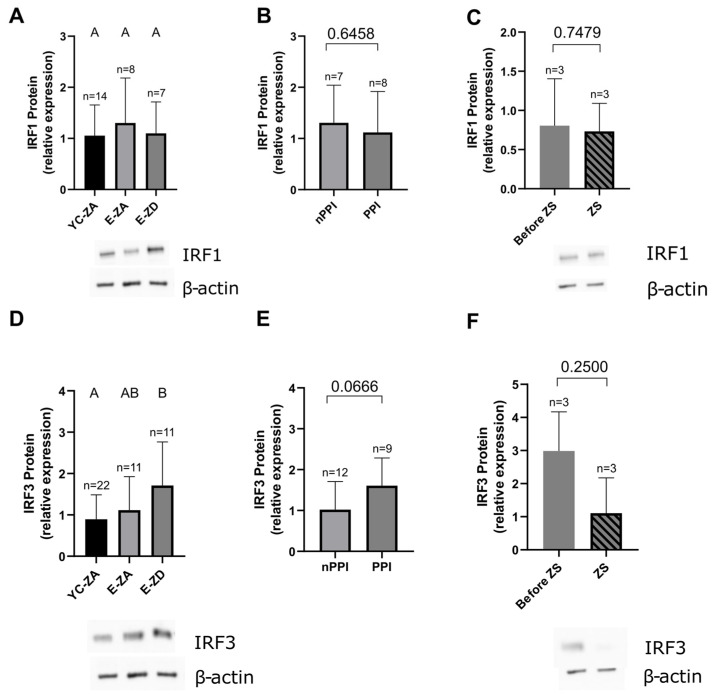
IRF1 (**A**–**C**) and IRF3 (**D**–**F**) protein expression in unstimulated PBMCs, measured by Western blot, normalized to β-actin and to the reference sample. IRF1 expression in (**A**) zinc-adequate young controls (YC, n = 14), zinc-adequate elderly (E-ZA, n = 8), and zinc-deficient elderly (E-ZD, n = 7)—YC vs. E-ZA: *p* > 0.9999; YC vs. E-ZD: *p* > 0.9999; E-ZA vs. E-ZD: *p* > 0.9999; (**B**) the elderly not taking PPIs (nPPI, n = 7) and those who do (PPI, n = 8)—nPPI vs. PPI: *p* = 0.6458; (**C**) E-ZD (n = 3) who received daily oral supplementation with 10 mg of zinc aspartate (ZS) for approximately 7 days (7.4 ± 1.9 days)—before ZS vs. ZS: *p* = 0.7479. IRF3 expression in (**D**) zinc-adequate young controls (YC, n = 22), zinc-adequate elderly (E-ZA, n = 11), and zinc-deficient elderly (E-ZD, n = 11)—YC vs. E-ZA: *p* > 0.9999; YC vs. E-ZD: *p* = 0.0238; E-ZA vs. E-ZD: *p* = 0.3222; (**E**) the elderly not taking PPIs (nPPI, n = 12) and those who do (PPI, n = 9)—nPPI vs. PPI: *p* = 0.0666; (**F**) E-ZD (n = 3) who received daily oral supplementation with 10 mg of zinc aspartate (ZS) for approximately 7 days (7.4 ± 1.9 days)—before ZS vs. ZS: *p* = 0.2500. Data are shown as means + SD. An outlier test (ROUT 1%) was performed on all data sets and outliers were removed (IRF3: YC n = 1, E-ZA n = 1, nPPI n = 2). Data sets with n < 8 were tested by the Shapiro–Wilk test and data sets with n ≥ 8 by the D’Agostino and Pearson test for normality. Data sets in (**A**,**B**,**D**,**E**) were considered unpaired and data in (**C**,**F**) paired, since the same subjects were analyzed before and after supplementation. For unpaired normally distributed data, we used the unpaired *t* test to compare two data sets (**B**,**E**). For unpaired non-normally distributed data, we used the Kruskal–Wallis test followed by Dunn’s multiple comparisons test for multiple data sets (**A**,**D**). For paired normally distributed data, the paired *t* test was used (**C**) and for the non-normally distributed data, the Wilcoxon test was used (**F**). Statistical significance is defined as *p* < 0.05. Means not sharing any letter in (**A**,**D**) are significantly different (*p* < 0.05). Representative Western blots are shown under (**A**,**C**,**D**,**F**).

IRF1 is an important regulatory protein in the IFN-γ signaling and TH1 polarization process [33,34]. In response to zinc, it shows context-dependent changes in expression [12,30]. In our experiments, we did not find a significant difference between young controls and zinc-adequate and zinc-deficient elderly participants (*p* > 0.9999 in all comparisons) (Figure 7A), nor a significant change in expression following zinc supplementation (*p* = 0.7479) (Figure 7C). PPI intake showed no significant impact on the IRF1 expression (*p* = 0.6458) (Figure 7B).

IRF3 is well studied in its role in interferon signaling, in its active, phosphorylated form [35]. However, we aimed to analyze its basal expression and found an unexpected pattern—the expression was significantly higher in the zinc-deficient elderly compared to the young control group (*p* = 0.0238), with the zinc-adequate elderly not significantly differing from either group (YC vs. E-ZA: *p* > 0.9999; E-ZA vs. E-ZD: *p* = 0.3222) (Figure 7D). Zinc supplementation led to a non-significant reduction in expression (*p* = 0.2500) (Figure 7F). Regarding PPI intake, the expression was higher in PPI patients, albeit only bordering on statistical significance (*p* = 0.0666) (Figure 7E). These results suggest an inverse correlation between zinc status and IRF3 expression, contrary to current evidence in the literature [31].

Considering our small sample size, studies with more participants are necessary to further explore the correlation between zinc status and the expression of the transcription factors IRF1 and IRF3.

## 3. Discussion

The same mechanisms that are necessary for the survival and reproductive ability in adults are the ones detrimental at advanced age—a circumstance not foreseen by evolution [36]. Both ill elderly and healthy centenarians showcase high levels of inflammation—how it affects individuals depends on environmental and genetic factors, which either promote robustness or frailty [36]. The concept of inflammaging describes this phenomenon, whereas immunosenescence is a broader term, encompassing the totality of changes in the aging immune system [2,36,37]. Mechanisms of immunosenescence are found in the most common age-related diseases such as neurodegenerative, cardiovascular, and metabolic diseases, rheumatoid arthritis, and cancer [38]. The elderly are more susceptible to infectious diseases, such as influenza and COVID-19, with higher associated mortality. Notably, effects of aging and zinc deficiency seem to overlap, raising the question of whether these are mutually conditional [5]. In addition, polypharmacy—a phenomenon very common in the elderly population—is an important risk factor for zinc deficiency since it can affect appetite, gastrointestinal, and renal function [21]. Proton pump inhibitors, commonly prescribed as comedication in polypharmacy patients, increase the risk of infections [39] and show a negative effect on IFN-γ production [24,27] in vitro. This raises the question of whether improving zinc status could mitigate some of the immunological consequences of both aging [5] and use of proton pump inhibitors.

Zinc homeostasis is strictly regulated, buffering the response to fluctuations in dietary zinc, and complicating the identification of reliable zinc biomarkers [40,41,42]. Serum and plasma zinc concentrations—representing less than 0.2% of total body zinc—remain the most commonly used indicators and can be used interchangeably [40,41,42]. Serum zinc concentration tends to respond to acute changes in intake [40,41,42], although homeostatic adaptations in absorption and excretion may prevent sustained increases with long-term high intake [40,43].

A significant correlation between age and serum zinc concentration has been well documented in the literature: concentrations peak at 18–25 years, gradually decline during adulthood, and drop noticeably after 65–70 years [44].

However, studies conducted exclusively in healthy elderly people reported mean serum zinc concentrations (95.8 ± 24.3 μg/dL, at 72 ± 7 years [45]) comparable to those of the young adults in our study (84.5 ± 10.3 μg/dL), and considerably exceeding the values we measured in slightly older hospitalized elderly participants (75.4 ± 11.0 μg/dL, at 82 ± 5 years). This suggests that zinc status correlates with the overall health status of the elderly and not solely with their age. Indeed, serum zinc correlates inversely with CRP in healthy adults aged 55–70 with baseline normal zinc status [43].

In our study, no correlation between age and serum zinc was observed within either group (young controls or elderly), consistent with previous studies in healthy elderly people [46]. Nevertheless, the significant difference in serum zinc between young and elderly groups aligns with the broader literature [17]. However, a study on healthy individuals with age spanning from 20 to 70 found no significant differences between the age groups regarding serum zinc concentration [47], suggesting that the compromised health status of hospitalized elderly participants in our cohort may have influenced our findings. Screening healthy elderly participants for immunogerontological studies that aim to analyze age as a sole factor in the changing immune system is a long-standing topic, with SENIEUR criteria being one of the earliest examples [48]. However, the question arises as to whether healthy elderly participants are representative of the average elderly individual [49]. The reverse question also arises—how applicable are our conclusions to the broader, non-hospitalized elderly population? The exclusion criteria applied in this study—current use of antibiotics or immunosuppressive medication, presence of solid tumors, hematologic malignancies, or autoimmune diseases at the time of sample collection—were chosen to minimize the influence of acute infections or hematologic alterations on the study outcomes. However, the effects of other medications and comorbidities were not systematically assessed. Therefore, further studies involving elderly participants from different settings and systematic reviews of such studies are required to grasp the overarching effects of aging, as well as to differentiate between effects of certain medications and comorbidities.

We observed a significant correlation between dietary zinc intake and serum zinc concentrations (*p* = 0.0014) using the food frequency questionnaire (FFQ) by Trame et al. [32]. Nonetheless, evidence in the literature on this topic remains inconsistent. Some studies describe a significant correlation [40], while others report a weak correlation [47] between dietary zinc intake and serum zinc levels. No significant changes in serum zinc were observed after dietary restriction for 20–24 weeks or zinc supplementation for 12 weeks [50], even though significant effects of altered dietary zinc intake on cytokine production, T-cell distribution, and homeostasis were shown. Similarly, increased dietary zinc intake in healthy adults over 4 weeks failed to elevate serum zinc levels, despite reducing DNA damage and oxidative stress markers [51]. These examples demonstrate the relevant effect of dietary zinc intake on the immune system, although this is not always reflected in changes in serum zinc concentrations.

Generally, zinc supplementation reliably increases serum zinc in adult and elderly individuals [13,17,31,40,52], and in animal models [53,54]. Individuals with low to moderate zinc status show the strongest response to supplementation [40]. In our study, short-term daily supplementation with Unizink^®^ 50 (10 mg per day) for 7.4 ± 1.9 days resulted in a significant increase in serum zinc concentration (*p* = 0.0436).

Zinc supplementation modulates immune responses depending on the context. Clinically, zinc supplementation reduces the duration of acute respiratory tract infections, including viral infections such as COVID-19 [5]. At the cellular level, supplementation increases antioxidant capacity, reduces inflammatory and oxidative stress markers [13], and enhances IL-2 production [17] in elderly participants. In healthy zinc-deficient vegans and vegetarians, zinc supplementation enhanced IFN-α production in response to viral stimulation after 2 weeks [31]. Supplementation also lowers basal IL-6 secretion while increasing LPS-stimulated IL-6 production [11], thus modulating this pro-inflammatory cytokine in a context-dependent manner. In addition, increases in IL-10—a crucial anti-inflammatory cytokine—and TNF-α—a pro-inflammatory cytokine—were shown in stimulated cells of zinc-supplemented adults [11]—further underlining the immunomodulatory role of zinc.

Aging is associated with impaired T-cell function and altered subset distributions, contributing to impaired cellular immunity in elderly individuals [5,38]. Supplementation in healthy adults aged 55–70 increased the T-helper/CTL ratio after 6 months of supplementation [43], suggesting improved balance between helper and cytotoxic T-cell responses. In healthy elderly participants (69.8 ± 5.1 years), daily supplementation for 7 weeks resulted in a significant decrease in CD25+ T-cells, which may reflect reduced activation or a normalization of overactive immune responses [52]. Conversely, supplementation in hospitalized elderly individuals led to a significant increase in IL-2 production after 6 days [17], indicating enhanced T-cell function.

IFN-γ, as the main cytokine of the Th1 response, plays a major role in the cell-mediated immunity, contributing to the defense against intracellular pathogens and immunosurveillance of infected or malignant cells. The effect of zinc on IFN-γ production varies depending on the immune context. In mixed lymphocyte culture—where IFN-γ secretion indicates the severity of the immune reaction and thus the severity of GvHD—zinc reduces the IFN-γ production, indicating an immunosuppressive/regulatory role [55]. Moreover, zinc’s influence on IFN-γ production depends on whether lymphocytes are exposed to novel versus previously encountered antigens, showcasing the fine modulatory effect of zinc rather than a purely immunosuppressive one [12]. Conversely, other studies using cell culture models demonstrate that zinc increases IFN-γ production in stimulated cells [9,10]. In adults, zinc supplementation does not elevate basal IFN-γ release, but enhances its production upon stimulation [11]. Our findings support this: post-supplementation IFN-γ levels remained mostly undetectable in unstimulated samples (data not shown, comparable to unstimulated controls shown in reference [11]), but increased significantly upon stimulation in whole-blood samples, compared to baseline (*p* = 0.0083), suggesting a regulatory effect of the zinc supplementation on the immune response.

Interestingly, we did not observe the same effect of zinc supplementation on IFN-γ production in PBMC samples. One possible explanation lies in the experimental design. PBMC samples were diluted in cell culture medium to achieve a cell density of 1 × 10^6^ cells/mL. The medium was prepared with 10% fetal calf serum (FCS) containing a consistent, serum zinc concentration. It is very plausible that, during the 48 h of incubation in this medium, the effects of zinc-deficient serum conditions in vivo on IFN-γ production were masked. This could explain why the pronounced differences between young adults and elderly participants—both zinc-adequate and zinc-deficient—remained significant, whereas more subtle differences between the two elderly groups could not be detected. In contrast, whole-blood experiments, which involve minimal dilution and retain much of the patient’s native serum environment, may be less affected by this masking effect, as evidenced by the significant effect of in vivo zinc supplementation on IFN-γ production. Thus, the lack of significant results in PBMC samples does not necessarily negate the zinc effect, but rather reflect the limitation of the PBMC culture model itself. In the context of zinc-dependent immune effects, whole-blood assays may be considered more physiologically relevant, and the two models should therefore not be weighted equally when interpreting results.

The question of whether these effects are sustainable with long-term zinc supplementation remains unanswered by our study due to its design. Previous research has shown that zinc supplementation for 7 weeks leads to a significant increase in both serum zinc concentration [52] and IFN-γ production [11], suggesting that similar effects might be observed with longer supplementation. It is important to note that long-term zinc supplementation carries a risk of hypocupremia and related complications [56], which is why supplementation is generally not recommended for periods longer than 3 months. Nevertheless, it has been shown that zinc and copper levels return to baseline within 3 months after the end of supplementation [56].

With abundant evidence suggesting beneficial effects of zinc supplementation in the elderly population, the question arises as to whether only zinc-deficient elderly individuals should be supplemented. Since zinc status assessment is not yet widely validated, would it be more practical to administer zinc supplementation broadly? It has been observed that individuals with low to moderate zinc status respond significantly to the supplementation, whereas it has minimal effects in individuals with high baseline zinc [40]. Additionally, excessive zinc can suppress cytokine production [4] and may induce hypocupremia [56]. These findings are especially relevant when considering supplementation in zinc-adequate individuals. Therefore, further studies with precautions such as regular copper monitoring are needed to adequately assess this question.

Proton pump inhibitors are some of the most commonly prescribed medications, used to treat a variety of gastric acid-related disorders and as a comedication with NSAIDs such as ibuprofen and diclofenac [39]. Although considered generally safe, new information about adverse effects continues to emerge. There is conflicting evidence regarding the correlation between PPI use and increased risk of COVID-19 infection and severe outcomes [39]. Additionally, some studies suggest an increased risk of pneumonia, gastrointestinal, and other infections [39]. Although the correlation with the infections is often attributed to the elevation in gastric pH via PPI [39]—the primary mechanism of this drug class—increasing evidence points to effects on the immune system at the cellular level, notably by impairing the function of neutrophils, macrophages [57], and T-cells [27]. To explore this further, we examined whether IFN-γ production is impacted by PPI use in vivo. Previous studies showed that incubation of cells with PPI reduces the production of IFN-γ [24,27] by inhibiting the transcription factor pCREB via a shift in the intracellular zinc [27]. In contrast, in our study, we found no significant association between PPI use and IFN-γ production in elderly hospitalized participants. However, this absence of effect might be related to the previously described limitation of our experimental design—incubation of cells in a medium with consistent, zinc-adequate conditions—which could have masked potential zinc-dependent differences in IFN-γ levels, despite PPI intake significantly altering markers of zinc status and thus theoretically expected to influence IFN-γ production.

Given that zinc plays an important role in IFN-γ regulation, we also examined the effect of PPI use on the zinc status of our elderly participants. Since PPIs are known for impairing the absorption of magnesium and calcium [39], it is worth investigating whether these pharmaceuticals impact the absorption of zinc. Indeed, we found significantly lower serum zinc concentrations in patients taking PPIs (*p* = 0.0008). We also observed that zinc deficiency occurred significantly more often in patients using PPIs, as confirmed by a chi-square test (*p* = 0.0118). The relative risk of 1.86 (95% CI 1.14–3.20) indicates that PPI users have nearly twice the risk of zinc deficiency compared to non-users. In addition, we report significantly lower dietary zinc intake in patients taking PPIs (*p* = 0.0206). Nausea and abdominal pain are some of the known short-term adverse effects of PPIs [39]. However, since the questionnaire assesses long-term eating habits, it is unlikely that nausea alone, as a PPI side effect, explains these findings. The question arises as to whether PPI use leads to reduced appetite in the long term—an aspect insufficiently explored in the current literature.

Taken together, our results suggest that PPI use is associated with impaired zinc status, as reflected in both serum zinc measurements and dietary intake assessments. The causality of this correlation remains to be elucidated. Although we did not observe changes in IFN-γ production in vivo associated with PPI use, further investigation in clinical settings is needed to clarify the mechanisms suggested by previous in vitro findings.

We wondered how these processes could be explained at a molecular level. We analyzed ZIP8 in unstimulated PBMCs using Western blot, to assess whether its expression might correlate with the zinc status of our participants. Although we did not find a significant difference between different groups, an increase in ZIP8 expression post supplementation was observed, but did not reach statistical significance. While analyzing supplementation effects in participants taking PPIs, we saw a significant increase in ZIP8 in a small sample size of three participants. It has been previously shown that PPIs suppress the ZIP8 expression in vitro [27], so it would be interesting to hypothesize that zinc supplementation may be able to reverse that. Although there is ample evidence that ZIP8 influences the intracellular zinc content and thus modifies cytokine expression and immune processes, there is not much evidence about the causality between zinc status in humans and ZIP8 expression on a cellular level. Studies involving zinc chelation and in vitro supplementation are needed to assess the effect of extracellular zinc on the expression of ZIP8, as well as human supplementation studies on larger sample sizes.

Additionally, we assessed exploratorily the expression of transcription factors IRF1 and IRF3 in unstimulated PBMCs, as potential downstream indicators of zinc- and IFN-γ-related immune modulation. Previous studies showed that IRF1 is regulated by zinc [12,30] and that it builds a positive feedback loop with IFN-γ—both by being induced by IFN-γ and by in turn further stimulating the production of IFN-γ, thus playing an important role in the polarization towards the Th1 response [12,30,33,34]. In our experiments, we did not find any significant difference in expression according to the zinc status of our participants, their PPI intake, nor in response to zinc supplementation. IRF3 is also an important interferon-regulating protein, especially well studied in its role in the IFN-α and IFN-β pathways. However, it is also activated by IFN-γ and plays an important role in the transcription of genes and expression of proteins downstream of IFN-γ and modulates the effect of IFN-γ on intracellular pathogens in a dependent manner [35,58,59]. Our group has recently shown a zinc-mediated upregulation of IRF3 resulting in elevated IFN-α production in young zinc-deficient vegans and vegetarians [31]. In this current study, IRF3 expression seems to rise with diminishing zinc status—zinc-deficient elderly participants show significantly higher IRF3 levels compared to young controls. Elderly participants taking PPIs show a trend towards higher IRF3 expression which borders on significance. There is a trend toward reduced IRF3 expression post supplementation—however, with only three samples available for this analysis, our results remain inconclusive. It needs to be clarified whether zinc supplementation affects the IRF3 expression in hospitalized elderly people in a different way than in the young healthy adults, and whether these results remain consistent in larger sample sizes.

The question remains as to how the experimental design affected protein levels in non-stimulated PBMCs. In this case, no incubation was performed; PBMCs were diluted in zinc-containing medium and immediately prepared for Western blot analysis by lysis and storage at −20 °C. Nevertheless, could even short-term, uniform exposure to a zinc-containing extracellular environment have masked differences in zinc-dependent protein expression within the cells?

In future research, it would be interesting to further analyze the role of zinc status, for example, by correlating the IFN-γ response post vaccination with the markers of zinc status. Additionally, long-term observational studies tracking zinc status and the incidence of infections would provide valuable insights. Long-term intervention studies involving zinc supplementation would require regular zinc and copper monitoring to prevent the above-mentioned toxicity due to excessive zinc intake.

## 4. Materials and Methods

### 4.1. Study Design and Dietary Zinc Intake Assessment

The study was conducted from April 2023 to April 2024. This study was reviewed and approved by the Institutional Ethics Committee of the Medical Faculty of RWTH Aachen University (young controls: EK023/05; elderly subjects: EK206/09). The research conforms to the principles of the Declaration of Helsinki.

Elderly participants (n = 78) were recruited by the study nurse from the Department of Geriatric Medicine at Franziskus Hospital in Aachen. All elderly participants provided informed written consent before sample collection. Criteria for inclusion in the study were age over 65 (mean age 82.2 ± 5.4 years), no antibiotic or immune-modulating medication, no immunosuppressive medication, no solid tumors, no hematologic malignancies, and no autoimmune diseases at the moment of sample collection, as well as no delirium and no dementia, as to not impair the ability to give informed consent. Dietary habits were assessed orally by the study nurse via a food frequency questionnaire and the adjusted zinc diet score (AZDS) was calculated [32] using the Zink-App [60]. Morning blood samples were collected regardless of fasting status, alongside routine clinical blood draws, thus avoiding additional venipuncture. Consistent with previous studies [17], participants with either AZDS below 113 points or serum zinc concentration under 70 μg/dL were considered zinc-deficient and therefore eligible for immediate zinc supplementation. These participants received the zinc supplement Unizink^®^ 50 (Köhler Pharma GmbH, Alsbach-Hähnlein, Germany), as clinically indicated. Post-supplementation blood samples were collected after approximately 7 days (7.4 ± 1.9 days) in 25 participants. Due to the end of hospital stay or laboratory delays, post-supplementation samples of 11 participants were not obtained. The same experimental regimen was applied before and after supplementation. Because the amount of blood collected and cell count varied greatly among elderly individuals, not all experiments could be performed on all samples. The exact study design is shown in Figure 1.

Young control participants (n = 115) were recruited via informal outreach among student groups and employees of the university hospital, laboratories, and other facilities. Potential participants were informed about the study and screened for the following inclusion criteria: omnivorous diet, age between 18 and 40 (mean age 24.3 ± 3.4 years), no acute or chronic diseases, and no regular medication except for oral contraception in females. Blood samples were collected at varying times of the day and independently of fasting status. Participants received the link to the zink-app [60] where they could fill out the same food frequency questionnaire used in the elderly group. Participants were asked to create a screenshot of their zinc score results and submit it. Participants were excluded from the study if they did not provide zinc score results (n = 3) and in case of zinc deficiency, based on AZDS or serum zinc assessment (n = 4).

### 4.2. Serum Zinc Measurement

Blood samples were collected in 9 mL serum Monovettes (Sarstedt, Nümbrecht, Germany). These were left for a minimum of 30 min at room temperature to allow for clotting. The Monovettes were then centrifuged at 1841× *g* for 10 min. The supernatant was then collected—2 mL of undiluted serum was stored in 2 mL tubes at −20 °C. In young control samples, 1 mL of serum was equally diluted with deionized water and stored in 15 mL tubes at 4 °C until measurement (storage guidelines in [61]). In elderly samples, two aliquots of equally diluted serum were stored in 15 mL tubes at 4 °C until the measurement. One aliquot was measured as soon as possible to determine participants’ eligibility for zinc supplementation. The second aliquot (pre-supplementation sample) was measured in the same run as the post-supplementation sample, to minimize the impact of inter-assay variability on the assessment of zinc supplementation effects on serum zinc concentration. The zinc concentration measurement was conducted via flame atomic absorption spectrometry using the AAnalyst 800 (Perkin-Elmer, Springfield, IL, USA) as previously described [31]. Consistent with previous studies [17], serum zinc concentrations below 70 μg/dL were considered zinc-deficient.

### 4.3. Whole-Blood Stimulation Assay

Blood samples were collected in 7.5 mL Lithium-Heparin Monovettes (Sarstedt) and diluted 1:10 with RPMI 1640 medium (Sigma Aldrich, Burlington, MA, USA), containing 10% calf serum (Capricorn Scientific, Ebsdorfergrund, Germany), 2 mM L-glutamine (Sigma Aldrich), and 1% Penicillin–Streptomycin (10,000 U/mL of Penicillin, 10 mg/mL of Streptomycin) (Sigma Aldrich). For each participant, one sample containing 0.25% PHA-L (Genaxxon bioscience, Ulm, Germany) and one control sample were incubated at 37 °C, 5% CO_2_ for 48 h. Thereafter, the supernatant was collected and stored for IFN-γ quantification at −20 °C.

### 4.4. PBMC Isolation and Incubation

Blood samples were collected in 7.5 mL Lithium-Heparin Monovettes (Sarstedt) and diluted equally with Dulbecco’s phosphate-buffered saline (1× PBS, Sigma Aldrich). This was then carefully layered onto an equal volume of Ficoll Lymphocyte Separation Medium (Capricorn Scientific) in 50 mL tubes and centrifuged at 600× *g* for 20 min at room temperature without brake. The buffy coat was harvested and washed three times with Dulbecco’s phosphate-buffered saline (1× PBS, Sigma Aldrich). Cells were adjusted to a final concentration of 1 × 10^6^ cells/mL using the RPMI 1640 medium (Sigma Aldrich), containing 10% calf serum (Capricorn Scientific), 2 mM L-glutamine (Sigma Aldrich), and 1% Penicillin–Streptomycin (10,000 U/mL of Penicillin, 10 mg/mL of Streptomycin) (Sigma Aldrich). For each participant, 2 samples of 1 × 10^6^ PBMCs were plated onto the 24-well plate—one sample containing 0.25% PHA-L (Genaxxon Bioscience) and one as the control. Both samples were incubated at 37 °C, 5% CO_2_ for 48 h. Thereafter, the cells were centrifuged and supernatants for IFN-γ quantification were stored in 2 mL tubes at −20 °C.

### 4.5. IFN-γ ELISA

Supernatants from whole blood and PBMCs were diluted in Assay Diluent (BD Biosciences, San Jose, CA, USA). IFN-γ concentration was quantified using OptEIA ELISA (BD Biosciences) according to the manufacturer’s protocol. Absorbance was measured using the Spark microplate reader (Tecan, Männedorf, Switzerland).

### 4.6. Western Blot

A total of 2 × 10^6^ PBMCs were centrifuged at 300× *g* for 5 min. The cell pellet was kept on ice and resuspended in 100 µL of lysis buffer containing 65 mM Tris–HCl (pH 6.8), 2% (*w*/*v*) SDS, 1 mM sodium orthovanadate, 26% (*v*/*v*) glycerol, 1% (*v*/*v*) β-mercaptoethanol, and 2% proteinase inhibitor cocktail (Boster Biological Technology, Pleasanton, CA, USA). This mixture was sonicated for 10 s using the Vibra Cell sonicator (Sonics & Materials Inc., Newtown, NY, USA), incubated for 3 min at 95 °C, and stored at −20 °C until further use.

Protein concentration was determined using a BCA assay prior to electrophoresis. A standard curve was generated by serial dilution of 2 mg/mL of bovine serum albumin (BSA) (PanReac AppliChem, Darmstadt, Germany) in buffer containing 65 mM Tris–HCl (pH 6.8), 2% (*w*/*v*) SDS, and 26% (*v*/*v*) glycerol. To each well of the 96-well plate, 150 µL of Pierce™ 660 nm Protein Assay Reagent (Thermo Fisher Scientific, Rockford, IL, USA) was added, followed by either 10 µL of the serial dilution samples, 10 µL of the blank (lysis buffer), or 5 µL of sample lysates. After shaking for 1 min, the plate was incubated at room temperature (RT) for 5 min. The absorbance was then measured using the Spark microplate reader (Tecan, Crailsheim, Germany).

Protein concentrations were calculated based on the standard curve after blank subtraction. Loading volumes were then calculated to deliver 20 µg of protein per gel well.

An amount of 1 µL of 0.5% (*w*/*v*) bromophenol blue was added to each sample lysate as a loading dye. An amount of 3 µL of the prestained protein ladder (New England BioLabs, Frankfurt am Main, Germany) and sample lysates were loaded onto a 10% SDS-PAGE gel. Electrophoresis was performed at 170 V for approximately 1 h.

The separated proteins in the gel were transferred to a nitrocellulose blotting membrane at 100 V. Successful transfer was confirmed by staining the membrane with Ponceau S (PanReac AppliChem, Darmstadt, Germany). The membrane was blocked with 5% powdered milk in TBS-T (containing 0.02 M Tris [pH 7.6], 0.15 M NaCl; 0.1% (*w*/*v*) Tween-20) for 1 h at RT on a horizontal shaker, then washed three times with TBS-T.

The membrane was incubated with a 1:500 solution of monoclonal rabbit primary antibodies (β-actin #4967, IRF1 #8478, and IRF3 #4302; all from Cell Signaling Technology, Leiden, The Netherlands; Zip8 #20459-1-AP, Proteintech, Planegg-Martinsried, Germany) in TBS-T containing 5% (*w*/*v*) BSA overnight at 4 °C on a roller. The next day, the membrane was washed three times in TBS-T and then incubated with the 1:2000 solution of secondary antibody (#7074, Cell Signaling Technology, Leiden, The Netherlands) in TBS-T with 5% (*w*/*v*) powdered milk for at least 2.5 h at RT on a roller.

After washing three times with TBS-T, the membrane was incubated with 3 mL of Westar Antares (Cyanagen, Bologna, Italy). The membrane was then carefully wrapped in one layer of clear film and imaged using LAS 3000 (Fujifilm Lifescience, Düsseldorf, Germany). The protein ladder was detected using digitize and precision modes, and the protein bands were detected using chemiluminescence and increment modes at different intensities.

The images were saved in TIFF format and analyzed using ImageJ 1.54d/Java 1.8.0_345 (64-bit) software (NIH, Bethesda, MD, USA). The density of target proteins was normalized to β-actin, and then to the reference sample, prepared from PBMCs of a young healthy control participant at the beginning of the study and included in each blot.

### 4.7. Statistical Analysis

Data were analyzed using GraphPad Prism 8.0.1. Outliers were removed prior to any statistical testing (ROUT method, Q = 1%) and data sets were assessed for normality using the D’Agostino and Pearson test. For comparisons of data before and after zinc supplementation, a paired *t*-test was used for normally distributed data, and a Wilcoxon test was used for non-normally distributed data. When comparing any other two data sets, an unpaired *t*-test (normal distribution) or a Mann–Whitney test (non-normal distribution) was performed. When comparing three or more data sets, ordinary one-way ANOVA with Holm–Šídák’s multiple comparisons test, assuming a single pooled variance, was performed for normally distributed data, and the Kruskal–Wallis test with Dunn’s multiple comparisons test was performed for non-normally distributed data. A two-sided chi-square test was conducted to assess the association between PPI use and zinc deficiency. Correlations were assessed using linear regression analysis. In all statistical tests, *p* < 0.05 was considered statistically significant. All values are presented as mean ± standard deviation.

## Data Availability

The datasets generated and/or analyzed during the current study are not publicly available but are available from the corresponding author on reasonable request.

## Abbreviations

The following abbreviations are used in this manuscript:

PPIs	Protein pump inhibitors
AZDS	Adjusted zinc diet score
YCs	Young controls
E	Elderly
ZA	Zinc-adequate
ZD	Zinc-deficient
ZS	Zinc supplementation
PBMCs	Peripheral blood mononuclear cells
